# Computer-Assisted Assessment of the Interaction Between Arousals, Breath-by-Breath Ventilation, and Chemical Drive During Cheyne-Stokes Respiration in Heart Failure Patients

**DOI:** 10.3389/fphys.2022.815352

**Published:** 2022-02-10

**Authors:** Gian Domenico Pinna, Roberto Maestri

**Affiliations:** Laboratory for the Study of Ventilatory Instability, Department of Biomedical Engineering, Montescano Institute - IRCCS, Istituti Clinici Scientifici Maugeri, Montescano, Italy

**Keywords:** hyperpnea, central apnea, ventilatory overshoot, ventilatory instability, state instability

## Abstract

Transient increases in ventilation induced by arousal from sleep during Cheyne-Stokes respiration in heart failure patients are thought to contribute to sustaining and exacerbating the ventilatory oscillation. The only possibility to investigate the validity of this notion is to use observational data. This entails some significant challenges: (i) accurate identification of both arousal onset and offset; (ii) detection of short arousals (<3 s); (iii) breath-by-breath analysis of the interaction between arousals and ventilation; (iv) careful control for important confounding factors. In this paper we report how we have tackled these challenges by developing innovative computer-assisted methodologies. The identification of arousal onset and offset is performed by a hybrid approach that integrates visual scoring with computer-based automated analysis. We use a statistical detector to automatically discriminate between dominant theta–delta and dominant alpha activity at each instant of time. Moreover, a statistical detector is used to validate visual scoring of K complexes, delta waves or artifacts associated with an EEG frequency shift, as well as frequency shifts to beta activity. A high-resolution (250 ms) state-transition diagram providing continuous information on the sleep-wake state of the subject is finally obtained. Based on this information, arousals are automatically identified as any state change from sleep to wakefulness lasting ≥2 s. The assessment of the interaction between arousals and ventilation is performed using a breath-by-breath, case-control approach. The arousal-associated change in ventilation is measured as the normalized difference between minute ventilation in the case breath (i.e., with arousal) and that in the control breath (i.e., without arousal), controlling for sleep stage and chemical drive. The latter is estimated by using information from pulse oximetry at the finger. In the last part of the paper, we discuss main potential sources of error inherent in the described methodologies.

## Introduction

Moderate-to-severe Cheyne-Stokes respiration with central sleep apnea (CSR-CSA)—an abnormal breathing pattern characterized by rhythmic rises and falls in ventilation interspersed with periods of central apnea or hypopnea—affects a large proportion (≈40%) of patients with heart failure and reduced ejection fraction, and is associated with increased morbidity and mortality (Bitter et al., 2011; Oldenburg et al., 2016).

Cheyne-Stokes respiration with central sleep apnea predominantly occurs during stage 1 (N1) and 2 (N2) non-rapid eye movement (NREM) sleep (Hanly et al., 1989). During these stages, it is also accompanied by a high number of arousals from sleep (Hanly et al., 1989; Pinna et al., 2018), which mostly occur during or at the beginning of the rising phase of cyclic hyperpneas (Pinna et al., 2018).

Although there is general agreement that instability in the chemoreflex control of ventilation is the primary underlying mechanism of CRS-CSA (Cherniack and Longobardo, 1973; Khoo et al., 1991; Javaheri and Dempsey, 2007; Khoo, 2010; Sands and Owens, 2016), the complex and interacting mechanisms mediating breathing instability are not yet fully understood. One important example is the interaction between chemoreflex-mediated instability and the so-called “state-driven instability” (Khoo et al., 1991).

It has been proposed that arousals from sleep occurring during the hyperpneic phase of CSR-CSA provide a transient extra-drive to respiratory and upper airway muscles, thereby causing a transient amplification of the chemoreflex-mediated hyperpnea (Yumino and Bradley, 2008; Khoo, 2010; Javaheri and Dempsey, 2013; Sands and Owens, 2016). This ventilatory overshoot, in turn, would promote a greater hypocapnia, leading to a prolongation of the following apnea (Xie et al., 1994; Khoo et al., 1996). Thus arousals, by transiently increasing ventilation, would contribute to sustaining and exacerbating CSR-CSA. The validity of this model, however, remains to be determined. Assessing model validity would therefore provide new insights into the pathophysiologic mechanisms underlying CSR-CSA. Indeed, addressing gaps in our understanding of the role played by arousals in ventilatory instability has recently been proposed as one of the research priorities for patients with CSR-CSA that may have therapeutic implications (Orr et al., 2021).

A major difficulty in validating the model is that it is not possible to establish a direct causal link between arousals and ventilatory changes using traditional experimental methods. Hence, a possible alternative approach is to explore the validity of the model using observational data (i.e., polysomnographic recordings during CSR-CSA), seeking indirect evidence supporting causality. This approach, however, entails some significant challenges: (i) accurate identification of both arousal onset and offset (state transitions are often characterized by complex and variable electroencephalographic patterns, making traditional visual identification of state changes difficult and subjective); (ii) detection of arousals <3 s in duration (they may have a functional significance but are not included in traditional visual scoring); (iii) breath-by-breath assessment of the interaction between arousals and ventilation (given that arousal may be much shorter than the hyperpnea, a high resolution is needed); (iv) adequate control for important confounding factors (e.g., chemical drive, sleep stage).

In this paper we report on our work in developing computational methods to address these challenges.

## Materials and Methods

### Signals

The developed methodologies require the use of high-quality, standard polysomnographic (PSG) recordings including: electrocardiogram (ECG), thoraco-abdominal movements by means of uncalibrated inductance plethysmography, nasal airflow with a nasal pressure transducer, oronasal airflow with a thermistor, oxygen saturation by pulse oximetry with a finger probe, electroencephalogram (EEG) (derivations: O1-M2, O2-M1, C3-M2, C4-M1, F3-M2, and F4-M1), electrooculogram (EOG) (derivations: E1-M2 and E2-M1), electromyogram (EMG) (chin and anterior tibialis muscles), and body position.

As a preliminary step, sleep studies are scored according to the recommendations of the American Academy of Sleep Medicine (AASM) (Berry et al., 2012, 2015). Scoring information as well as raw signals are then transferred to a dedicated workstation for subsequent analysis using in-house developed software.

### Identification of Arousals

#### General Concepts

The term “arousal” conventionally denotes a temporary change from a state of sleep to a state of wakefulness (ASDA Report, 1992; Berry and Gleeson, 1997; White and Younes, 2012), or, more elegantly, a temporary intrusion of wakefulness into sleep (Halász et al., 2004). To accurately identify these state changes, we use a hybrid approach that includes both visual and computer-based analysis of PSG recordings, thereby exploiting the strengths of each. On the one hand, information derived from visual analysis of unequivocal EEG activity is processed by computer algorithms to classify EEG activity during periods of less clear activity. On the other hand, information derived from visual analysis of dubious EEG patterns is processed by computer algorithms to objectively validate and refine this tentative scoring. The overall procedure includes five key steps that are detailed below.

Given that both CSR-CSA and arousals predominantly occur in stages N1 and N2 (Hanly et al., 1989), the methodologies we have developed so far focus on these periods of sleep.

#### Methodology

##### Step 1: Visual Scoring of Relevant Electroencephalogram/Electrooculogram Features and Events

###### Background

Visual scoring is still considered the gold standard in the identification of EEG/EOG features and events. The reliability of the scoring depends on several factors, including the signal-to-noise ratio, the experience of the scorer, the duration of the event and the number of EEG derivations and additional signals (e.g., ECG, respiration) used in the analysis (Bonnet et al., 2007).

###### Purpose

The purpose of this step is to visually score EEG/EOG features and events that are relevant to the dentification of arousals.

###### Procedure

**FIGURE 1:**
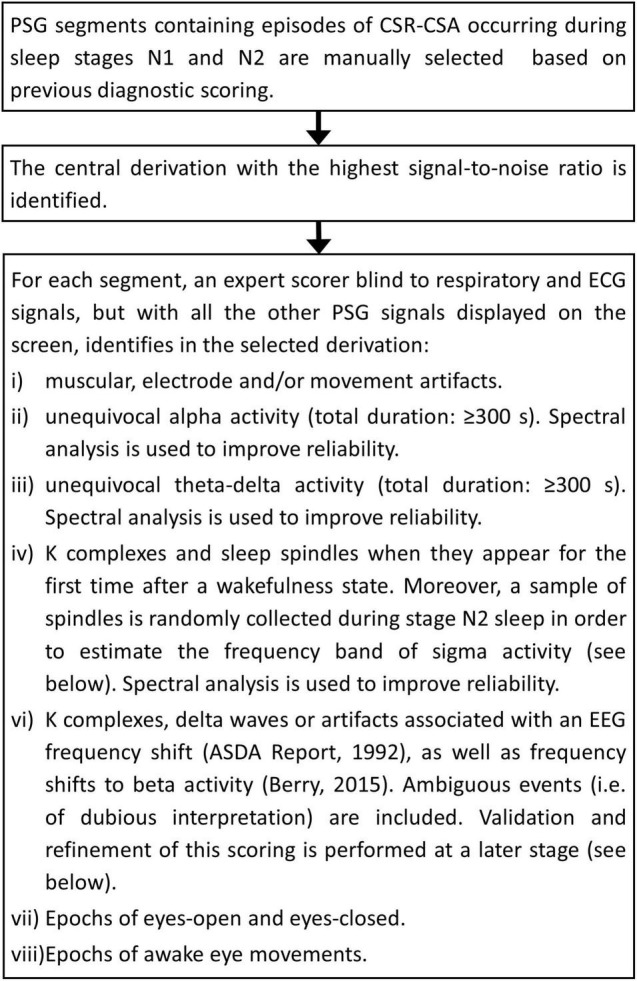
Visual scoring of relevant EEG/EOG features and events: flowchart diagram.

The flowchart of the procedure is shown in Figure 1. A few representative examples of the scoring process are given in Supplementary Figures 1–3. The overall analysis is reviewed by an independent scorer and, in case of disagreement, polysomnographic tracings are re-examined jointly and an agreement reached.

##### Step 2: Automatic Estimation of Patient-Specific Frequency Boundaries of Theta–Delta, Alpha, Sigma, and Beta Activity

###### Background

Computing the power of the EEG signal in relevant frequency bands (theta–delta, alpha, sigma, beta) using conventional fixed frequency boundaries is problematic because these boundaries typically change from patient to patient (Penzel and Conradt, 2000).

###### Purpose

The purpose of this step is to estimate individual frequency boundaries to be used in the design of the digital filters of the statistical discriminator (see below).

###### Procedure

**FIGURE 2:**
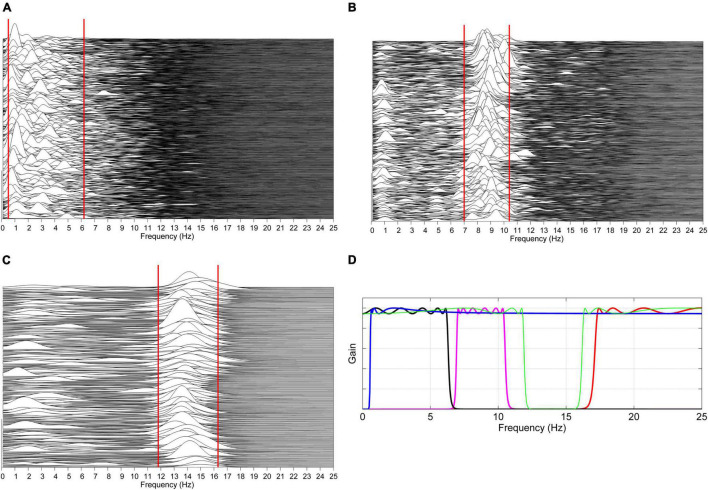
Automatic estimation of patient-specific frequency boundaries of theta–delta, alpha, sigma, and beta activity. **(A)** The spectra of previously identified unequivocal theta–delta epochs are computed over consecutive 2 s periods overlapped by 50%. For each spectrum, the lower and upper limit of the region between 0.5 and 7 Hz containing 80% of the signal power are automatically determined, and the 5th and 95th percentile of, respectively, collected lower and upper limits are considered as frequency boundaries of theta–delta activity for that specific patient (solid red lines). The same procedure is carried out on unequivocal alpha epochs **(B)** and sleep spindles **(C)**, considering the region of frequencies between 6 and 13 Hz and between 11 and 17 Hz, respectively. Patient-specific lower and upper limits for beta activity are set at +1 Hz the estimated upper limit of sigma activity and at 30 Hz, respectively. Based on the estimated boundaries, four corresponding zero-phase, bi-directional Chebyshev band-pass filters are automatically designed **(D)**. Data shown in plots panels **(A–D)** are from a male heart failure patient (age: 62 years; New York Heart Association class: III; left ventricular ejection fraction: 33%; dominant central sleep apnea; apnea–hypopnea index: 32.6/h; central apnea index: 9.8/h).

The procedure is illustrated in Figure 2.

##### Step 3: Automatic Discrimination Between Dominant Alpha and Dominant Theta–Delta Activity (Statistical Discriminator)

###### Background

Although arousals typically manifest as an abrupt shift in EEG frequency (ASDA Report, 1992), a more gradual shift from theta–delta to overt alpha activity, with progressive increase of the latter and/or periods of overlapped theta–delta and alpha activity, may also occur (Santamaria and Chiappa, 1987). These mixed patterns are similar to those commonly observed during the transition from wakefulness to NREM sleep, when moderate-amplitude theta–delta activity occurs at alpha disappearance (Santamaria and Chiappa, 1987; Ogilvie, 2001). Both phenomena may cause uncertainty and arbitrariness in the visual identification of the arousal onset and offset. Therefore, an objective continuous identification of the dominant EEG activity between alpha and theta–delta is crucial.

###### Purpose

The purpose of this step is to determine which of the two activities—alpha or theta–delta—is dominant at each instant of time (resolution: 250 ms).

###### Procedure

The identification of dominant EEG activity is based on the probability distribution of the power ratio between alpha and theta–delta fluctuations in the two conditions of unequivocal alpha activity and unequivocal theta–delta activity. Information on the distributions in these reliable conditions is then used to classify EEG activity when the identification of dominant activity is less certain.

**FIGURE 3:**
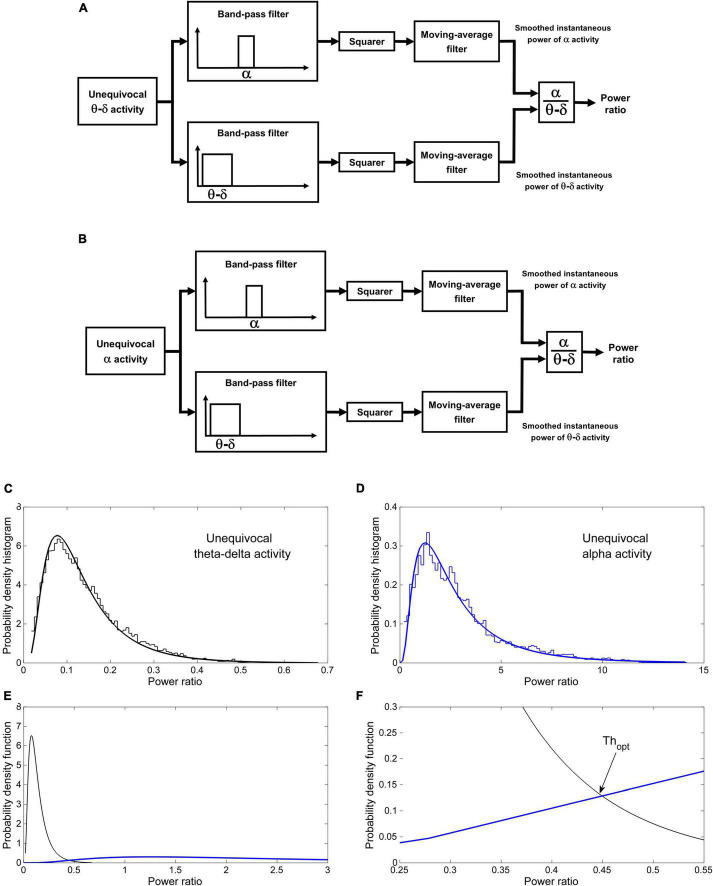
Automatic discrimination between dominant theta–delta and alpha activity: learning phase. The instantaneous alpha/theta–delta ratio (power ratio) during epochs of unequivocal theta–delta **(A)** and alpha activity **(B)** is computed by dividing the smoothed instantaneous power of alpha activity by the same power of theta–delta activity. The probability distribution of the power ratio in the two conditions is then estimated **(C,D)**. Finally, the optimal threshold Th_opt_ for discriminating between dominant alpha and dominant theta–delta activity is determined as the value of the power ratio at which the two distributions cross **(E)** [likelihood ratio = 1 (Albert and Harris, 1987)]. **(F)** Magnification of the intersection region. Data are from the same patient of Figure 2.

**FIGURE 4:**
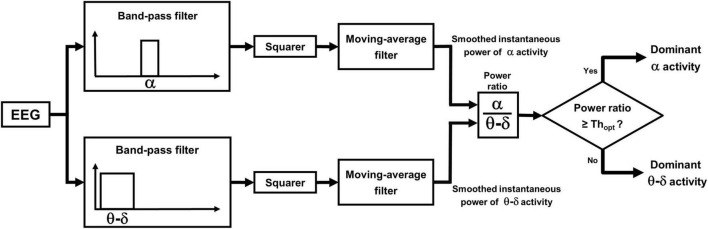
Automatic discrimination between dominant theta–delta and alpha activity: discrimination phase. The power ratio is re-computed across the whole length of all analyzed segments, excluding previously scored artifacts, awake eye movements and eyes-open. EEG periods with a power ratio ≥ Th_opt_ are automatically classified as dominant alpha activity, while those with a ratio < Th_opt_ are classified as dominant theta–delta activity (output of the statistical discriminator; time resolution: 0.25 s).

The procedure comprises two phases: a learning phase and a discrimination phase. In the learning phase the probability distributions of the power ratio are estimated by analyzing the epochs of unequivocal alpha and theta–delta activity previously identified by the scorer. The crossing point between the two distributions identifies a patient-specific threshold (Th_opt_) for discriminating between dominant alpha and dominant theta–delta activity. A detailed description is given Figure 3. In the discrimination phase, the estimated threshold is used to automatically classify the *overall* EEG recording into dominant alpha activity or dominant theta–delta activity (output of the statistical discriminator; time resolution: 0.25 s). Full details are given in Figure 4.

##### Step 4: Computer-Assisted Validation and Refinement of Visually Scored Electroencephalogram Frequency Shifts

###### Background

Because of the dominance of their low-frequency components, K complexes, delta waves and artifacts accompanied by an EEG frequency shift are inevitably misclassified by the statistical discriminator as dominant theta–delta activity. Moreover, the discriminator is not designed to detect frequency shifts to beta rhythms. For these reasons, the detection of these frequency shifts is based on traditional visual scoring. Sensitivity and reliability are well-known issues in scoring low-amplitude, fast-frequency shifts, particularly when the signal-to-noise ratio is low or short events are considered (Bonnet et al., 2007). Accordingly, in order to increase sensitivity without sacrificing reliability, ambiguous frequency shifts are included in the visual scoring process and a computer-assisted procedure is used to objectively confirm or reject all scored events and refine their beginning and end.

###### Purpose

The purpose of this step is to validate previous visual scoring of K complexes, delta waves or artifacts associated with an EEG frequency shift, as well as previous visual scoring of frequency shifts to beta activity. In the case of successful validation, their onset and termination is refined.

###### Procedure

**FIGURE 5:**
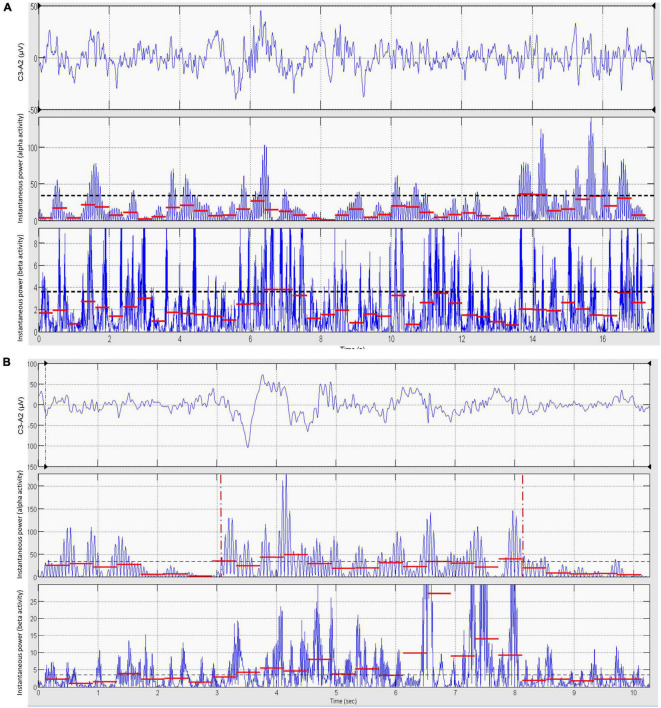
Computer-assisted validation and refinement of visually scored EEG frequency shifts. Using the output of the statistical discriminator as a guide, the scorer selects a baseline epoch (15–20 s) of dominant theta–delta activity as close as possible to the supposed frequency shift (**A,** upper panel). The EEG signal of this epoch is band-pass filtered in the alpha band and the mean value of the instantaneous power of alpha activity in consecutive 0.4 s segments computed (**A,** mid panel, red segments). The 95th percentile of the distribution of these values (dashed black line) is taken as the threshold for the detection of a significant increase in alpha activity during the subsequent validation of the suspected frequency shift. The same procedure is repeated for beta activity (**A,** lower panel, red segments). The scorer then selects the epoch containing the suspected frequency shift (**B,** upper panel). The EEG signal is filtered in the alpha and beta bands and the mean power of alpha and beta activity calculated as above (**B,** mid and lower panels, red segments). If at least five consecutive segments (i.e., 2 s) of alpha and/or beta activity have a mean power greater than the respective detection thresholds derived from the baseline epoch, the suspected frequency shift is considered as statistically significant and the onset and offset of the frequency shift are set by a computer algorithm at, respectively, the beginning and termination of the overall region of continuous significant increase in alpha and/or beta activity (**B,** mid panel, vertical red markers). If significance is not reached, testing is repeated in the other EEG derivations (ASDA Report, 1992; Berry et al., 2015). Data are from the same patient of Figure 2.

The procedure is illustrated in Figure 5.

##### Step 5: Automatic Construction of the State-Transition Diagram and Identification of Arousals

###### Background

Based upon reliability issues, the minimum duration required to score an arousal according to AASM recommendations is 3 s (ASDA Report, 1992; Bonnet et al., 2007; Berry et al., 2015). However, it has been suggested that shorter arousals may have functional significance and that computer-based methods may improve detection sensitivity and reliability (Martin et al., 1997; Bonnet et al., 2007; White and Younes, 2012). Because of similar reliability issues, the minimum amount of intervening sleep necessary to score independent arousals is 10 s (ASDA Report, 1992). Again, computer-based methods, being based on objective rules, would allow to improve reliability.

###### Purpose

The purpose of this step is to: (1) automatically construct a three-level, high-resolution, state-transition diagram providing continuous information on the sleep-wake state of the subject using information from both the statistical discriminator and visual scoring; (2) automatically detect arousals relaxing the 3 and 10 s rules.

###### Procedure

The state of the state-transition diagram is identified at each point in time (resolution: 250 ms) according to the following rules:

1. **Wakefulness** (W). This state is identified if at least one of the following conditions is present: (i) dominant alpha activity, (ii) K complex, delta wave or artifact associated with an EEG frequency shift, (iii) a frequency shift to beta activity, (iv) awake eye movements, (v) eyes open.
2. **NREM1**. This state is identified if a dominant theta–delta activity preceded by the same state or by a W state is present. NREM1 continues until the occurrence of a new W state or the appearance of a K complex or sleep spindle. It corresponds to traditional sleep stage N1.
3. **NREM2**. This state begins with the appearance of a K complex or sleep spindle and continues for as long as dominant theta–delta activity is present. NREM2 ends when a new W state begins. It corresponds to traditional sleep stage N2.

**FIGURE 6:**
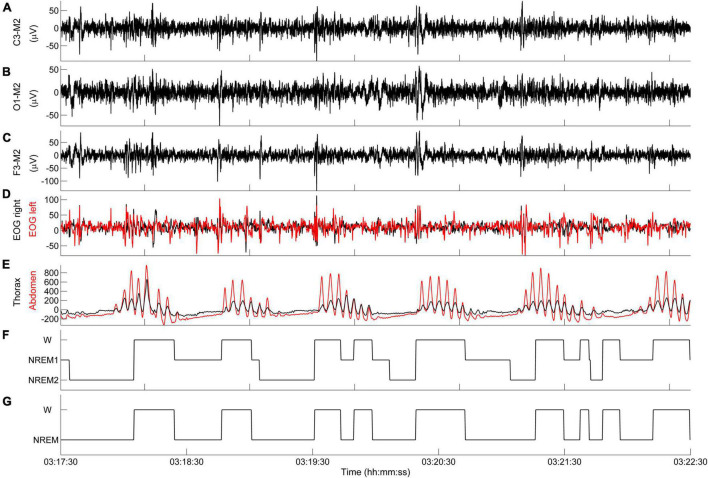
Representative example of state-transition diagram. **(A–E)** PSG tracings during an episode of CSR-CSA. **(F)** State-transition diagram. **(G)** Simplified state-transition diagram where NREM1 and NREM2 states are represented as a single NREM state. Data are from a male heart failure patient (age: 55 years; New York Heart Association class: III; left ventricular ejection fraction: 31%; dominant central sleep apnea; apnea–hypopnea index: 20.6/h; central apnea index: 7.1/h).

According to these definitions, the following state transitions are determined: (i) W → NREM1, (ii) NREM1 → W, (iii) NREM1 → NREM2, (iv) NREM2 → W. Only state transitions with a minimum length of 2 s are included in the diagram. To simplify the interpretation of results, the state-transition diagram is typically depicted as a two-level diagram (W, NREM). An example is given in Figure 6.

Arousals are automatically identified in the state-transition diagram as any state change from NREM1 or NREM2 to W lasting ≥2 s, with the beginning and end of each change marking the arousal onset and offset, respectively.

#### Experimental Findings

The basic methodology for the analysis of state transitions has been validated in 16 patients with heart failure and CSR during short-term (25 min), daytime PSG laboratory recordings in the supine position (Pinna et al., 2012), a condition that is often associated with the development of periodic breathing (La Rovere et al., 2017). Briefly, segments with unequivocal alpha and theta–delta activity were randomly divided into a training set and a testing set. Patient-specific frequency boundaries and the threshold of the discriminator were computed in the training set and then used in the testing set to automatically identify dominant alpha and theta–delta activity. The output of the automatic identification was compared point-by-point (5 ms resolution) with previous visual scoring of unequivocal alpha and theta–delta activity, and the percentage of points correctly classified by the discriminator was computed. We found that 99.1 ± 1.4 and 99.2 ± 1.1% of unequivocal alpha and theta–delta activity were correctly classified as dominant alpha and dominant theta–delta activity, respectively (Pinna et al., 2012).

Using the same daytime PSG recordings, we assessed the relationship between sleep-wake fluctuations, as described by the state-transition diagram, and apneic events (Pinna et al., 2014). The main findings of the study were that 92% of central apneic events were associated with a concurrent W → NREM → W transition, and that tidal volume, minute ventilation and inspiratory drive dramatically increased at the termination of the apnea almost synchronously with the occurrence of a NREM → W transition.

The methodology was then extended to nighttime recordings in order to accurately determine the time of occurrence and duration of arousals during apneic and hyperpneic phases of CSR-CSA in stages N1 and N2 (Pinna et al., 2018). Standard PSG was performed in 22 heart failure patients with dominant central sleep apnea, an apnea–hypopnea index of 39.5 ± 9.3/h and a central apnea index of 20.1 ± 9.8/h. Data segments including all episodes of CSR-CSA occurring during sleep stages N1 and N2 were interactively selected and the arousal onset and duration were determined using the methodology described above. We found that, on average, 27.5% of hyperpneic phases of CSR-CSA were not associated with an arousal, with a wide variation between patients (range: 2.1%, 57.4%). When present, hyperpneic arousals mostly occurred almost synchronously with the resumption of breathing after the apnea or within the first half of the hyperpnea, with the latter arousals about twice as frequent as the former. These arousals were occasionally followed by one or two shorter secondary arousals which likely represented a fragmentation of the preceding arousal.

### Assessment of the Interaction Between Arousals and Ventilation

#### General Concepts

An accurate assessment of the interaction between arousals and ventilation during CSR-CSA requires a breath-by-breath approach. This is due to two main reasons. First, the arousals occurring during the hyperpneic phases of CSR-CSA may be as short as a few seconds (Pinna et al., 2018); therefore, they may potentially affect ventilation in just one or two breaths. Second, the ventilatory response to arousals may change from breath to breath owing to the concurrent change in the inhibitory feedback from lung stretch receptors induced by the cyclic rise and fall in tidal volume (Kubin et al., 2006).

The assessment of the interaction can be performed using a case-control, computer-based procedure, comparing the ventilation in each breath concurrent with an arousal with that of a matched breath not concurrent with an arousal.

#### Methodology

##### Step 1: Estimation of Breath-by-Breath Ventilation

###### Background

Monitoring of respiratory activity during sleep is commonly carried out using uncalibrated respiratory inductance plethysmography (RIP). To estimate breath-by-breath ventilation, the rib cage and abdomen signals must first be calibrated to obtain a calibrated lung volume signal. We use the qualitative diagnostic calibration method, which is carried out during regular breathing and does not require connection to a spirometer or pneumotachograph (Sackner et al., 1989).

###### Purpose

The purpose of this step is to estimate breath-by-breath ventilation.

###### Procedure

The calibration factor is computed as the mean value of three calibration factors obtained in three randomly selected segments during regular breathing, and is then used to obtain a lung volume signal in relative units. Breath-by-breath minute ventilation ($\dot{V}$_I_) is finally computed as the ratio of breath-by-breath tidal volume to corresponding breath duration.

##### Step 2: Estimation of Oxygen Saturation at Carotid Chemoreceptors

###### Background

In the absence of arousal, the primary underlying mechanism of the periodic breathing of CRS-CSA is instability in the chemical regulation of breathing (Cherniack and Longobardo, 1973, 2006; Javaheri and Dempsey, 2007; Khoo, 2010). Accordingly, cyclic hyperpneas occurring during CSR-CSA are caused by the concurrent cyclic stimulation of chemoreceptors by hypoxia and hypercapnia resulting from the delayed effect of previous apnea or hypopnea.

Experimental observations and modeling studies indicate that carotid chemoreceptors exert a dominant influence on the development of periodic breathing (Smith et al., 2003; Khoo, 2010; Marcus et al., 2014). These receptors respond very rapidly to changes in arterial carbon dioxide (PaCO_2_) and oxygen (PaO_2_) tensions in the blood that perfuses them (Fidone and Gonzalez, 1986). During CSR-CSA, PaCO_2_ and PaO_2_ oscillate in phase opposition (Faber et al., 1990); therefore, the rise and fall in ventilation is nearly in phase with the rise and fall in PaCO_2_ at carotid chemoreceptors (Khoo, 2010), and nearly opposite in phase with the rise and fall in PaO_2_ at the same receptors. Because arterial PaO_2_ and oxygen saturation (SaO_2_) are in phase (Faber et al., 1990), the rise and fall in ventilation during CSR-CSA is also nearly opposite in phase with the rise and fall in SaO_2_ at carotid chemoreceptors. PaCO_2_ and SaO_2_ are monotonically inversely related (Gotoh et al., 1969; Khoo et al., 1991) and are linearly related to ventilation (Berger et al., 1977). Hence, the SaO_2_ signal provides a convenient surrogate marker for the cyclic activation and deactivation of carotid chemoreceptors during CSR-CSA.

Pulse oximetry (SpO_2_) is the recommended technique for non-invasive monitoring of SaO_2_ during PSG (Berry et al., 2015). The most suitable site for the SpO_2_ sensor during sleep studies aimed at investigating CSR-CSA, is the ear (Millar et al., 1990), as the oscillation of SpO_2_ is only slightly delayed relative to carotid bodies (Pinna et al., 2000). However, for practical reasons, monitoring of SpO_2_ is almost invariably performed placing the sensor at the finger. Because of the more distal position, changes in SpO_2_ at the finger are markedly delayed relative to carotid chemoreceptors (Maestri et al., 2019).

###### Purpose

The purpose of this step is to estimate the time course of oxygen saturation at carotid chemoreceptors using the SpO_2_ signal recorded at the finger. This is necessary to implement the next step of the procedure.

###### Procedure

**FIGURE 7:**
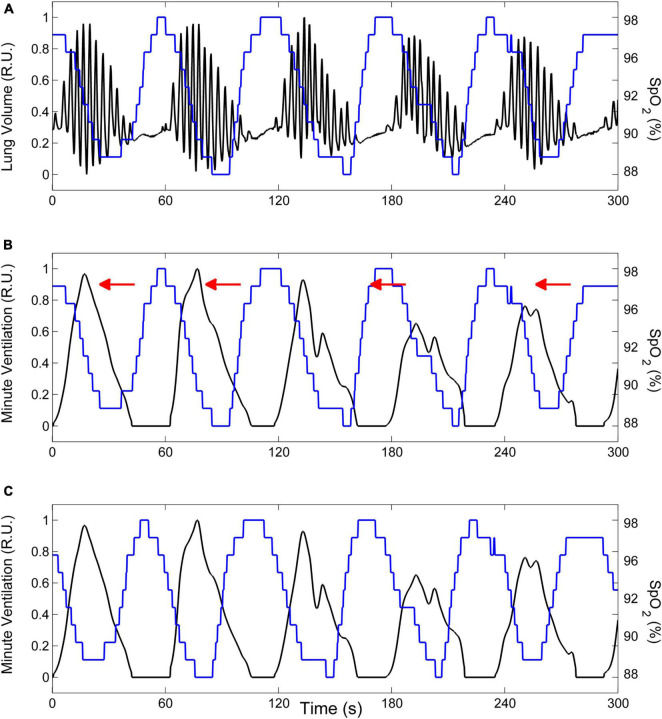
Example of the procedure to estimate the time course of oxygen saturation at carotid chemoreceptors. **(A)** Lung volume (black line) and oxygen saturation at the finger (SpO_2_, blue line) during CSR-CSA. **(B)** The lung volume signal is replaced by the corresponding instantaneous minute ventilation signal. **(C)** The estimate of oxygen saturation at carotid chemoreceptors is obtained by shifting the SpO_2_ signal recorded at the finger backward in time (as indicated by red arrows in panel **B**) until the antiphase condition between cyclic hyperpneas and oxygen saturation is met. R.U.: relative units. Data are from a male heart failure patient (age: 45 years; New York Heart Association class: III; left ventricular ejection fraction: 24%; dominant central sleep apnea; apnea–hypopnea index: 38.2/h; central apnea index: 23.5/h).

We estimate the time course of oxygen saturation at carotid chemoreceptors by shifting the SpO_2_ signal recorded at the finger backward in time, until the antiphase condition between cyclic hyperpneas and oxygen saturation is met. This condition is determined by finding the shift that maximizes the non-linear correlation coefficient between the SpO_2_ signal and the instantaneous minute ventilation signal during hyperpneas (Maestri et al., 2019). The latter is obtained using a previously developed method (Pinna et al., 2000). An example is given in Figure 7.

##### Step 3: Estimation of Arousal-Associated Changes in Ventilation

###### Background

Arousals are thought to contribute to the ventilatory instability of CSR-CSA by promoting a transient amplification of the hyperpnea, thereby leading to the prolongation of the subsequent apnea.

###### Purpose

The purpose of this step is to estimate the breath-by-breath, arousal-associated change (AAC) in ventilation in each patient.

###### Procedure

**FIGURE 8:**
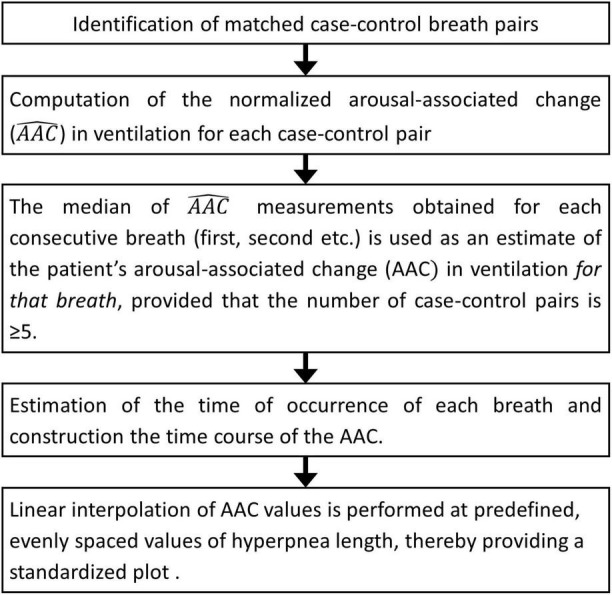
Estimation of breath-by-breath, arousal-associated change (AAC) in ventilation: flowchart diagram.

**FIGURE 9:**
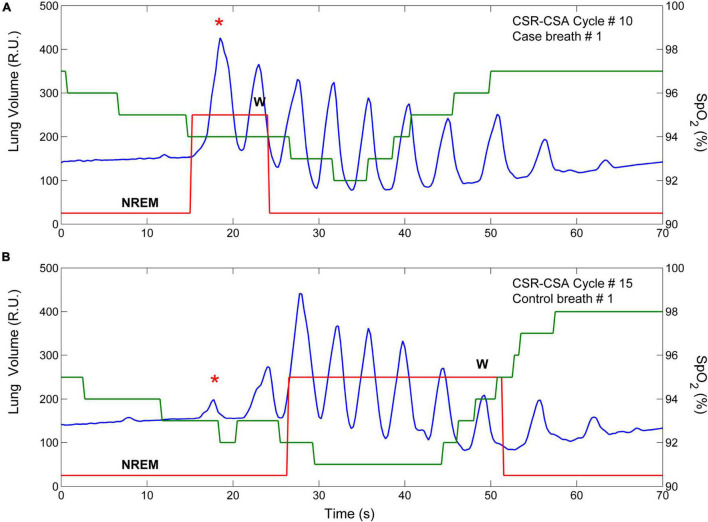
Example of breath-by breath case-control matching. The two panels show the lung volume (blue line), the state-transition diagram (red line), and the estimated oxygen saturation at carotid chemoreceptors (SpO_2_, green line) in two CSR-CSA cycles of the same patient. **(A)** An arousal occurred during the hyperpneic phase of the 10th CSR-CSA cycle, potentially affecting the first two breaths. **(B)** A matched control breath for the first breath was found in the 15th CSR-CSA cycle (closest cycle). By definition, mean SpO_2_ of the case and control breaths were similar (94 vs. 92.8%), as well as the duration of the hyperpneic phase (49 vs. 47 s). The sleep stage was the same (N2). R.U. = relative units. Data are from a male heart failure patient (age: 58 years; New York Heart Association class: III; left ventricular ejection fraction: 17%; dominant central sleep apnea; apnea–hypopnea index: 39.3/h; central apnea index: 23.9/h).

The flowchart of the procedure is shown in Figure 8. Details are given in the following. CSR-CSA cycles beginning with and/or followed by a hypopnea are excluded from this analysis to avoid ambiguity in the identification of the beginning and end of the hyperpnea. A breath of the hyperpneic phase of a CSR-CSA cycle is classified as “case” if there is a temporal overlap between an arousal and the breath. For each identified case, a search is made for a matched “control” breath in the closest CSR-CSA cycle, according to the following criteria: (i) same sequential position in the hyperpnea (first, second, third…); (ii) no concurrent arousal and ≥10 s from the arousal offset of a previous arousal, if any; (iii) same estimated mean oxygen saturation at carotid chemoreceptors ±1.5%; (iv) same sleep stage (N1 or N2); (v) same hyperpnea length ±15%. If a control is found (Figure 9), the normalized arousal-associated change ($\hat{A\,A\,C}$) in $\dot{V}$_I_ for that case-control pair is computed as the difference between the case breath and the control breath, normalized by the average $\dot{V}$_I_ in the absence of arousal during all hyperpneas, namely:


(1) $$\hat{A\,A\,C}\,{\left({\dot{V}}_{I}\right)}_{i\,j\,k}=\frac{\begin{array}{c} {\dot{V}}_{I}\,{\left(c\,a\,s\,e\,b\,r\,e\,a\,t\,h\right)}_{i,j}\\ -{\dot{V}}_{I}\,{\left(c\,o\,n\,t\,r\,o\,l\,b\,r\,e\,a\,t\,h\right)}_{i,k} \end{array}}{\begin{array}{c} A\,v\,e\,r\,a\,g\,e\,{\dot{V}}_{I}\,i\,n\,t\,h\,e\,a\,b\,s\,e\,n\,c\,e\,o\,f\\ a\,r\,o\,u\,s\,a\,l\,d\,u\,r\,i\,n\,g\,h\,y\,p\,e\,r\,p\,n\,e\,a\,s \end{array}}\times 100$$

where *i (i =* 1,2,3…N) is the progressive number of the breath, *j* (*j* = 1,2,…M) is the progressive number of the CSR-CSA cycle containing the case breath, and *k* (*k* = 1,2,…L) is the progressive number of the cycle containing the control breath. For instance, $\hat{A\,A\,C}\,{\left({\dot{V}}_{I}\right)}_{3,20,22}$refers to the case-control pair including the third breath of the 20th CSR-CSA cycle (case breath) and the third breath of the 22nd cycle (control breath). Hence, if $\hat{A\,A\,C}\,{\left({\dot{V}}_{I}\right)}_{3,20,22}$ = 50%, the increase in ventilation in the case breath relative to the control breath is equal to half the average ventilation during hyperpneas in the absence of arousals. This computation is repeated until all case-control pairs are identified in each patient across CSR-CSA cycles. The median of $\hat{A\,A\,C}$ measurements obtained for each consecutive breath (first, second, etc.) is then used as an estimate of the patient’s arousal-associated change (AAC) in $\dot{V}$_I_ *for that breath*, provided that the number of case-control pairs is ≥5.

**FIGURE 10:**
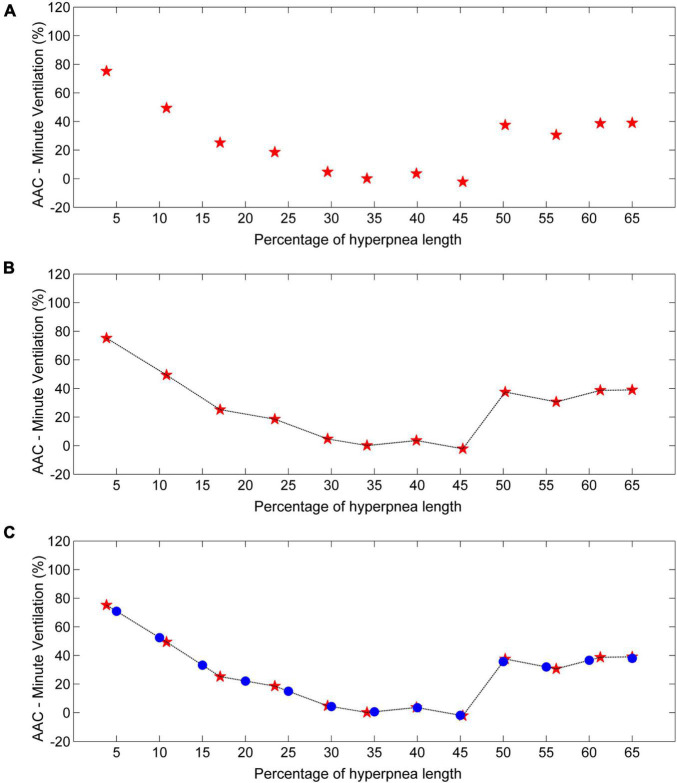
Example of estimation of the time course of the arousal-associated change (AAC) in minute ventilation. **(A)** AAC estimates (red stars) for each consecutive breath of the hyperpnea as a function of percentage of hyperpnea length. **(B)** Linear interpolation of AAC estimates (black line). **(C)** The AAC is re-computed (blue dots) at predefined, uniformly spaced hyperpnea times (5%, 10%, 15%, ….). Data are from a male heart failure patient (age: 62 years; New York Heart Association class: II; left ventricular ejection fraction: 36%; dominant central sleep apnea; apnea–hypopnea index: 52.3/h; central apnea index: 17.7/h).

Each time a new case-control pair is identified, the time of end-inspiration of the case breath is measured and expressed as a percentage of hyperpnea length. The median of the measurements obtained for each consecutive breath (first, second, etc.) is then taken as an estimate of the time of occurrence of that breath in that patient. Using this information, a description of the time course of the AAC in $\dot{V}$_I_ during the hyperpnea is easily obtained for each patient. A representative example is given in Figure 10A.

To compare the time course of the AAC in $\dot{V}$_I_ among patients, a linear interpolation of individual AAC values is performed (Figure 10B), and the AAC is re-computed at predefined, evenly spaced values of hyperpnea length (5%, 10%, 15%, …). The final result is a standardized plot describing the time course of the AAC in $\dot{V}$_I_ during the hyperpnea (Figure 10C).

##### Step 4: Estimation of the Time Course of Ventilation in the Absence of Arousal

**FIGURE 11:**
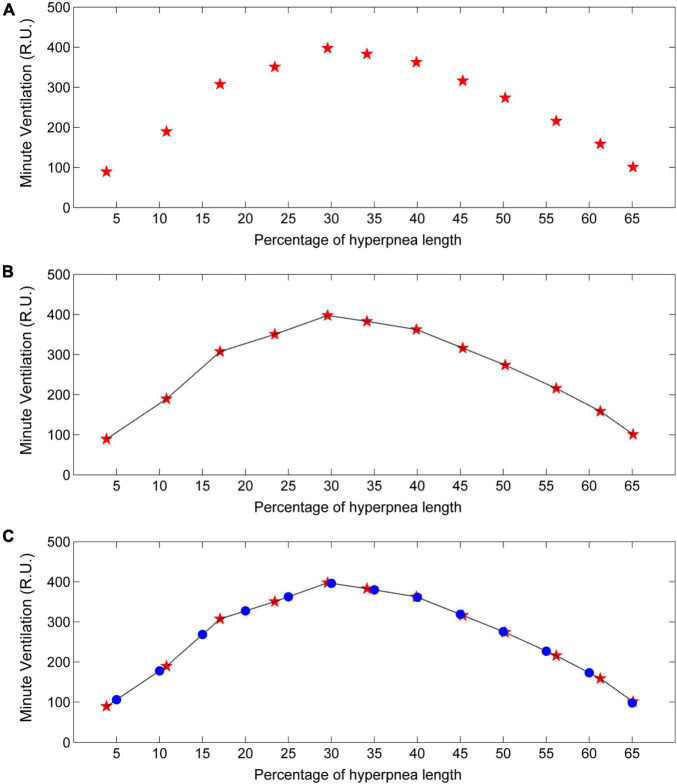
Example of estimation of the time course of baseline minute ventilation during hyperpneas. **(A)** Minute ventilation estimates (red stars) in the absence of arousal for each consecutive breath of the hyperpnea as a function of percentage of hyperpnea length. **(B)** Linear interpolation of the estimates (black line). **(C)** Minute ventilation is re-computed (blue dots) at predefined, uniformly spaced hyperpnea times (5%, 10%, 15%, ….). R.U. = relative units. Data are from the same patient of Figure 10.

Each time a new case-control pair is identified, the ventilation of the control breath is also recorded. The median of collected measurements for each consecutive breath (first, second, etc.) is taken as an estimate of the patient’s baseline ventilation (i.e., in the absence of arousal). Using again linear interpolation and standardization of the time scale, the estimate of its time course is finally obtained (Figure 11).

#### Experimental Findings

We recently used this methodology to assess the interaction between arousals and ventilation in 18 heart failure patients with dominant central sleep apnea, an apnea–hypopnea index of 40.4 ± 8.6/h and a central apnea index of 21.5 ± 10.0/h (Pinna et al., 2021). We found that: (1) arousals occurring during the hyperpneic phases of CSR-CSA were associated with a non-homogeneous change in ventilation characterized by a rapidly decreasing ventilatory overshoot at the beginning of the hyperpnea, followed by a tendency toward a slight ventilatory undershoot around its peak. (2) The presence of arousal during the hyperpnea was associated with a modest average increase in the length of the subsequent apnea, but there was a large inter-subject variability (range: -8.5%, +36.2%). (3) The incidence of early-hyperpneic arousals (i.e., those occurring at the beginning of the hyperpnea) and the arousal-associated change in ventilation around peak hyperpnea were strong and independent determinants of the change in apnea length (*R*^2^ = 0.78). Of note, the incidence of early-hyperpneic arousals ranged from 5 to 70%, indicating a large variation across patients.

### Critique of the Methods

The transition from W to NREM1 is represented in the state-transition diagram as an abrupt shift between the two states. This is a simplification of the sleep onset process, which is typically gradual. Indeed, before the disappearance of alpha activity and the appearance of a stable theta–delta activity, an irregular mixture of both rhythms often occurs (Santamaria and Chiappa, 1987; Ogilvie, 2001). Because such patterns have a broadband spectrum containing both oscillations, the output of the statistical discriminator tends to show a sequence of very short periods alternating between dominant alpha and dominant theta–delta activity. Therefore, in order to provide an interpretable diagram, we follow the rule to change the state of the state-transition diagram from W to NREM1 only when theta–delta activity is predominant for at least 2 s. The same rule is followed during the so-called attenuation periods, characterized by a dramatic decrease of EEG activity, so that the distinction between signal and noise is difficult (Santamaria and Chiappa, 1987; Ogilvie, 2001).

Despite the use of computer-assisted methods, the identification of arousal onset and offset is subject to some degree of error. A characteristic of this timing error, however, is its random nature. Therefore, since individual estimates are the median across all episodes of CSR-CSA occurring during sleep stages N1 and N2, the impact of measurement error on study results would be very low.

Matching for chemical drive in the case-control procedure (Figure 9) was performed using oxygen saturation as a surrogate marker for the cyclic activation and deactivation of carotid chemoreceptors, although these receptors are sensitive to both hypoxia and hypercapnia. Given that PaCO_2_ and SaO_2_ are monotonically inversely related, this surrogate would be adequate for case-control matching because what is needed in this procedure is to select case-control breath pairs with a similar level of chemical drive, not to know the value of the chemical drive itself.

## Conclusion

The application of the methodologies described above has significantly improved our understanding of the complex interaction between arousals and ventilation during CSR-CSA in heart failure patients. Three main conclusion emerge from our analyses. First, arousals are not essential for the propagation CSR-CSA, but when present they are associated with a longer apnea, thereby contributing to sustaining and exacerbating breathing instability. Second, this effect varies widely from patient to patient. Third, this variability is highly dependent on the incidence of early-hyperpneic arousals and the arousal-associated ventilatory change at peak hyperpnea. These findings suggest that the impact of arousals on CSR-CSA is highly patient dependent, and therefore pharmacological manipulation of the arousal threshold to prevent the occurrence of arousals may represent a therapeutic option to reduce/suppress CSR-CSA only in selected patients.

## Data Availability Statement

The original contributions presented in the study are included in the article/Supplementary Material, further inquiries can be directed to the corresponding author.

## Author Contributions

GP conceived the study and wrote the manuscript. RM conceived the study, developed the software, and critically reviewed the manuscript. Both authors contributed to the article and approved the submitted version.

## Conflict of Interest

The authors declare that the research was conducted in the absence of any commercial or financial relationships that could be construed as a potential conflict of interest.

## Publisher’s Note

All claims expressed in this article are solely those of the authors and do not necessarily represent those of their affiliated organizations, or those of the publisher, the editors and the reviewers. Any product that may be evaluated in this article, or claim that may be made by its manufacturer, is not guaranteed or endorsed by the publisher.
