# Supplementary material for: Computer-Assisted Assessment of the Interaction Between Arousals, Breath-by-Breath Ventilation, and Chemical Drive During Cheyne-Stokes Respiration in Heart Failure Patients
Source: Front Physiol. 2022 Feb 10;13:815352. doi: 10.3389/fphys.2022.815352 (PMC8867072; doi:10.3389/fphys.2022.815352)
Supplement: Supplementary file 1 [file Data_Sheet_1.PDF]

Computer-assisted assessment of the interaction between arousals, breath-by-breath ventilation and chemical drive during Cheyne-Stokes respiration in heart failure patients

Gian Domenico Pinna[1], Roberto Maestri[1]

Supplementary Material

Fig. S1-S3.

Representative examples of visual scoring of relevant EEG/EOG features and events. The name of each event is followed by its duration. Theta-delta: unequivocal theta-delta activity. Alpha: unequivocal alpha activity. K: K-complex. K-δ arousal: K complex or delta wave associated with an EEG frequency shift.

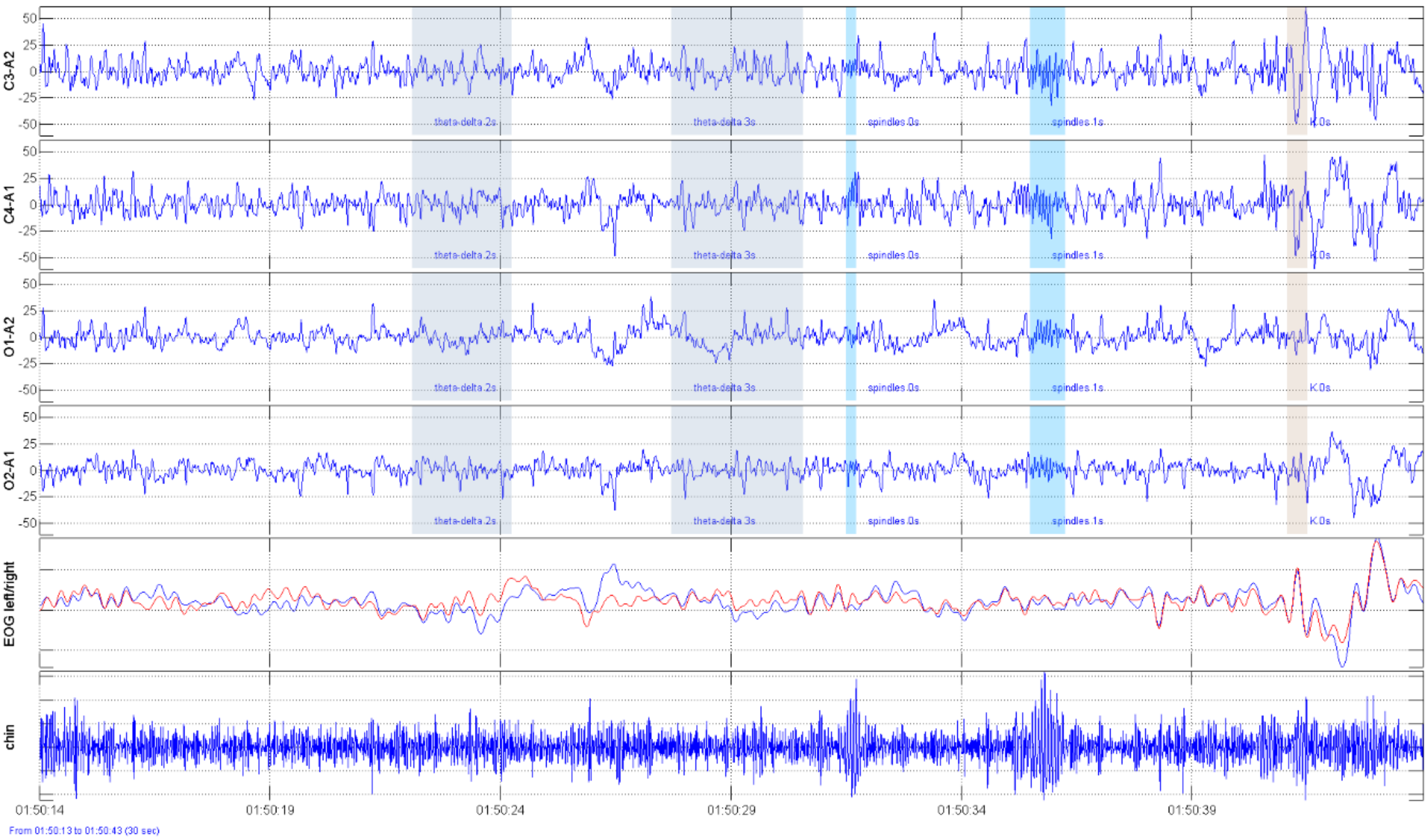

C3-A2
C4-A1
O1-A2
O2-A1
EOG left/right
chin
50
25
0
-25
-50
theta-delta 2s
theta-delta 3s
spindles 0s
spindles 1s
K 0s
01:50:14
01:50:19
01:50:24
01:50:29
01:50:34
01:50:39
From 01:50:13 to 01:50:43 (30 sec)

Figure S1

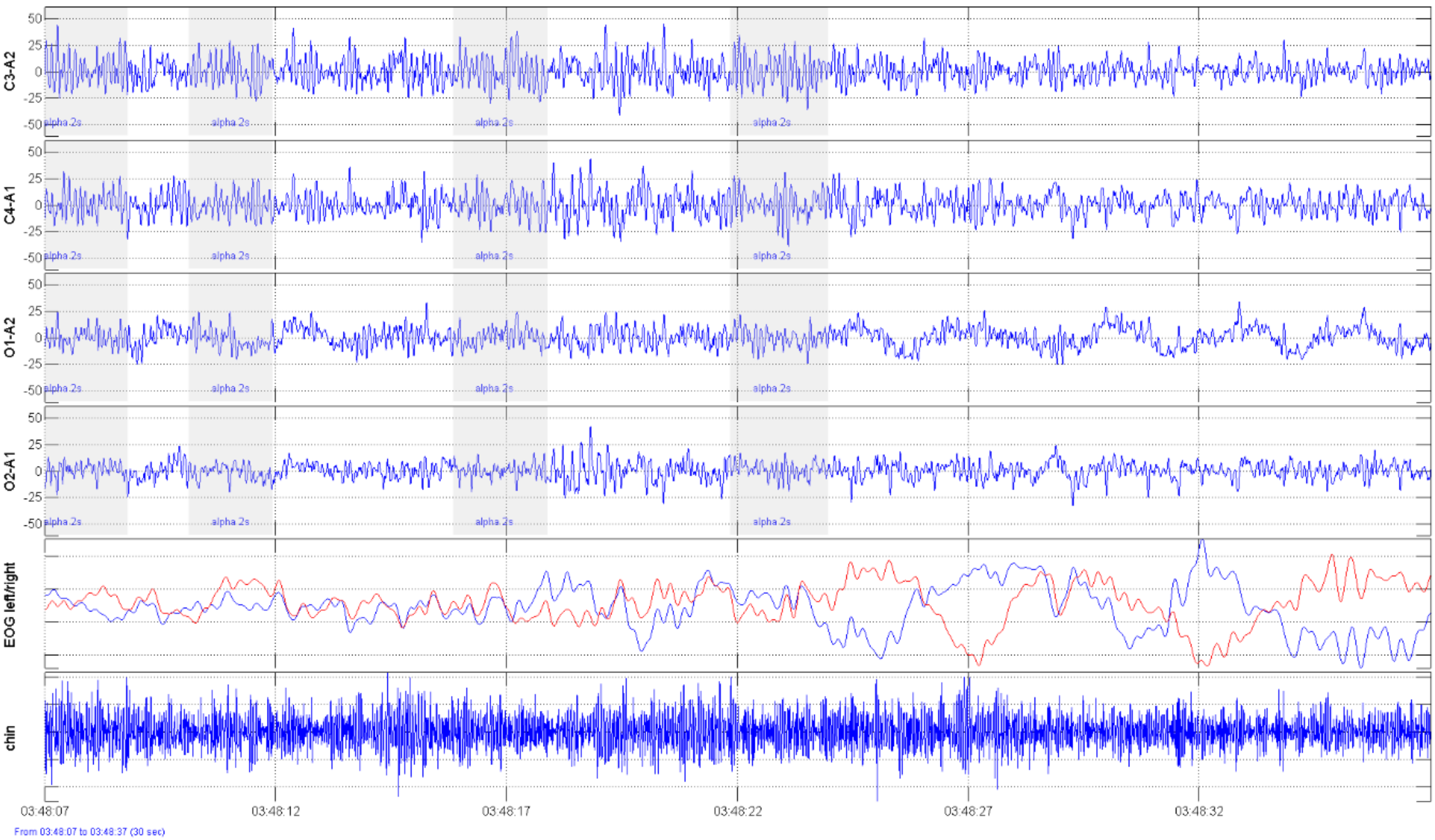
C3-A2
C4-A1
O1-A2
O2-A1
EOG left/right
chin
50
25
0
-25
-50
alpha 2s
03:48:07
03:48:12
03:48:17
03:48:22
03:48:27
03:48:32
From 03:48:07 to 03:48:37 (30 sec)

Figure S2

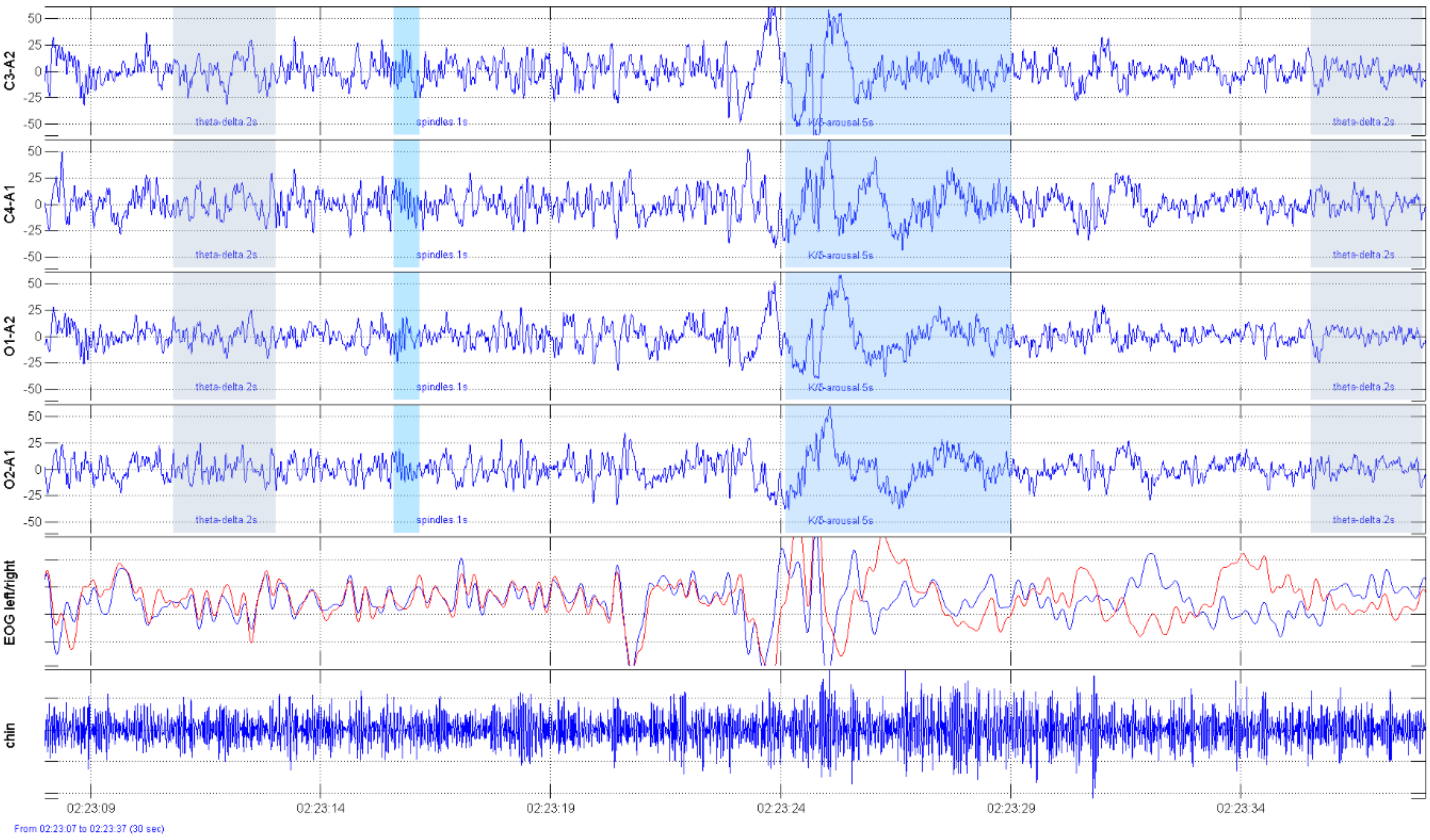

C3-A2
C4-A1
O1-A2
O2-A1
EOG left/right
chin
50
25
0
-25
-50
theta-delta 2s
spindles 1s
K/S-arousal 5s
02:23:09
02:23:14
02:23:19
02:23:24
02:23:29
02:23:34
From 02:23:07 to 02:23:37 (30 sec)

Figure S3

Notes on developed software

All the algorithms described in the paper have been developed using Matlab 8.3 (Natick, Massachusetts: The MathWorks Inc), a technical computing environment for high performance numeric computation and visualization. We will make our software and all information required to set up a replication of our system available to researchers interested in sharing research protocols with our group.
